# MS-DOCK: Accurate multiple conformation generator and rigid docking protocol for multi-step virtual ligand screening

**DOI:** 10.1186/1471-2105-9-184

**Published:** 2008-04-10

**Authors:** Nicolas Sauton, David Lagorce, Bruno O Villoutreix, Maria A Miteva

**Affiliations:** 1 INSERM, U648, 45 rue des Sts Peres, University Paris Descartes, 75006 Paris, France

## Abstract

**Background:**

The number of protein targets with a known or predicted tri-dimensional structure and of drug-like chemical compounds is growing rapidly and so is the need for new therapeutic compounds or chemical probes. Performing flexible structure-based virtual screening computations on thousands of targets with millions of molecules is intractable to most laboratories nor indeed desirable. Since shape complementarity is of primary importance for most protein-ligand interactions, we have developed a tool/protocol based on rigid-body docking to select compounds that fit well into binding sites.

**Results:**

Here we present an efficient multiple conformation rigid-body docking approach, MS-DOCK, which is based on the program DOCK. This approach can be used as the first step of a multi-stage docking/scoring protocol. First, we developed and validated the Multiconf-DOCK tool that generates several conformers per input ligand. Then, each generated conformer (bioactives and 37970 decoys) was docked rigidly using DOCK6 with our optimized protocol into seven different receptor-binding sites. MS-DOCK was able to significantly reduce the size of the initial input library for all seven targets, thereby facilitating subsequent more CPU demanding flexible docking procedures.

**Conclusion:**

MS-DOCK can be easily used for the generation of multi-conformer libraries and for shape-based filtering within a multi-step structure-based screening protocol in order to shorten computation times.

## Background

Recent advances in the human genomics and proteomics projects have significantly contributed to the large number of macromolecular targets entering drug discovery programs. Along the same line, over 10 million organic compounds are presently available from chemical vendors and can be used in high throughput screening (HTS) experiments or *in silico *computations. However the escalating costs of both, experimental assays and hardware/software, highlight the need for development of novel approaches to assist rapid and efficient hit identification. Here we focus our attention on structure-based virtual ligand screening (SBVLS) methods [1-4] since they are known to be effective for library prioritization in the context of a drug discovery campaign or chemogenomics initiatives [5-7]. Numerous free or open-source VLS tools are available to academic groups [8,9] and such *in silico *methods play a major role in facilitating the identification of new lead compounds.

The ideal SBVLS method should predict both, the pose and the affinity of the ligands and be able to deal with flexibility [10]. Such tool does not exist today and trying to dock millions of molecules on thousands of targets with the available flexible docking methods eventually followed by real computations of binding affinities is out of reach to most research groups. In fact, one may wonder about the rational of docking/scoring millions of compounds with, for instance, highly CPU demanding stochastic methods (i.e., they may need several independent runs), into binding pockets for which maybe half (or more) of the molecules can not fit. Although complementary physical-chemistry features are of major importance in determining protein-ligand binding affinity, shape complementarity is also essential [11,12] indicating that rigid body docking programs should be revisited. Furthermore, the current trend is to use high-quality [13] target-focused libraries instead of huge compound collections in SBVLS or HTS projects as active compounds tend to be hidden by the noise of the database [14,15]. Clearly, in SBVLS projects, it seems reasonable to focus the chemical space of the compound libraries according to geometric restraints dictated by receptor-binding site [16] prior to dock/score all the compounds of a database into a binding pocket via flexible methods. A number of tools and protocols (i.e., multi-step SBVLS protocols) exploit this notion of rigid-body docking or at least use approaches that analyze the shape or the shape complementarity only, as a first step of a hierarchical procedure, to rapidly reduce the size of the compound collections prior to using more accurate and time consuming docking/scoring computations [16-21].

Several methods for performing shape complementarity search between a ligand and its receptor have been developed. One of the first programs for protein-small molecule interaction involving shape complementarity search by rigid body docking was DOCK, developed by Kuntz and co-workers [22]. The program DOCK generates a negative image of the receptor by making use of spheres that fill the binding pocket. The algorithm then attempts to superimpose the ligand atoms onto the centers of the spheres. A matching between the ligand and the receptor by a superimposition of ligands atoms onto the receptor surface was also employed in [23]. Other programs performing rigid-body docking between small molecules and proteins are FLOG [24], CLIX [25] and FRED [26]. This latter applies a Gaussian shape fitting function to optimize the contact surface between the ligand and the protein thereby allowing extremely fast rigid docking procedure.

Despite obvious limitations, rigid-body docking methods are interesting because they are much faster than the flexible docking algorithms and because significant noise can be generated with fully flexible docking of large collections. A common approach to improve rigid-body docking accuracy is to employ a library of compounds containing pre-generated multiple conformers for each ligand. Indeed, docking of an ensemble of multiple conformations of ligands into a receptor site with a modified version of DOCK demonstrated much better enrichment as compared to rigid docking with one single conformer per ligand [27]. In order to use efficiently rigid body methods, multi-conformer libraries have to be generated, with well established commercial packages [28,29] such as Corina/Rotate (Molecular Networks GmbH) (Gasteiger Research), OMEGA (Openeye Scientific Software), Catalyst [30] or with free tools like FROG [31].

Although rigid-body docking methods are attractive for hierarchical SBVLS drug discovery projects [16,19,32], these ones are under-exploited. In this paper we present the multiple conformation rigid-body docking filter MS-DOCK that is based on the program DOCK. Our protocol was tested on seven target proteins with different binding site properties for its ability to retrieve 65 known inhibitors in a library of 37970 drug-like compounds. The performance of MS-DOCK was additionally validated through a comparison with the commercial program OMEGA for multi-conformer generation and the program FRED for rigid-body docking (i.e., in this study we used FRED as a shape complementarity filter not for a full screening procedure). MS-DOCK was able to successfully decrease the initial compounds collection by 2- or 3-fold depending on the ligands size (if known) and binding pocket shape. Such a reduced compound collection focused on the pocket shape can then be used in a subsequent extensive flexible docking phase, either performed with DOCK or other tools. As a result, our rigid-body docking approach prioritizes the compounds by selecting only the molecules that have satisfactory shape complementarity with the receptor-binding pocket.

## Results

### MS-DOCK approach

In this work we describe an efficient approach MS-DOCK based on DOCK to rapidly screen a molecular database and to enrich the output collection in molecules having good shape complementarity with a given protein target binding site. The MS-DOCK approach involves two main steps: first, generating multiple 3D conformers for the flexible molecules present in the input chemical library, this step is performed with the in-house developed tool Multiconf-DOCK based on the systematic search approach available in DOCK5 [33]; second, all multiple conformations are rigidly docked with DOCK6 (see Methods for details) using the geometric match method on the rigid protein target.

#### Multiconf-DOCK: method and implementation

We developed the program Multiconf-DOCK for small molecule multi-conformer generation that requires as starting point the 3D structure of each input molecule in mol2 format. The method is based on a systematic search for ligand flexibility [34] as implemented in DOCK5. The 3D structure of the molecule is constructed starting from an anchor segment and the flexible parts are added via an incremental re-construction approach named "anchor-first". We used the multiple fragment option to consider several possible anchor fragments. Conformations were generated by rotating all single, non-terminal, acyclic bonds in specified increments. Using the torsion drive method [34] low energy dihedral values were tried for each torsion previously defined. In our implementation we increased significantly the exploration of the conformational space by enlarging the number of allowed positions. These changes were introduced because it was demonstrated that the low energy angle values as implemented in DOCK were not sufficient to reproduce the ligand-bound conformation as observed in experimentally determined protein-ligand complexes [16,32]. Thus for the single bonds between aromatic cycles and sp2 hybridized atoms, we allowed 6 positions with 45° rotation increments while the amide bond was kept rigid. The single bonds sp2-O.3/S.3 atoms (Tripos atom type) and aromatic cycle-O.3/S.3 atoms were also considered to be rotatable with 45° increments.

Multiconf-DOCK tool can efficiently sample different conformations of a ligand due to a RMSD cut-off defined by the user, as well as a RMSD comparison with an external conformation. To compute the RMSD between two different structures, both conformations are superimposed by a least-squares fit procedure [35]. The allowed conformations for each molecule are selected according to calculated energy involving the van der Waals and Coulomb terms with the Amber force field as implemented in DOCK5. The energy threshold, like the RMSD, can be defined by the user. In this study the maximal energy allowed to accept a conformer was set to be 25 kcal/mol, in addition an energy threshold of 25 kcal/mol between the initial single conformation and the generated one was permitted. For application in the subsequent rigid-body docking stage 2, we generated up to 50 conformers for each molecule present in the chemical library with a RMSD value of 1.0 Å. To write Multiconf-DOCK, we modified the program DOCK5 written in C++ while the RMSD and the energy filters as well as changes in the flexibility parameters mentioned above were additionally implemented. Multiconf-DOCK is operational on Linux and Mac OS X systems.

#### Assessment of multiple conformation generation by Multiconf-DOCK and OMEGA

**Figure 1:**
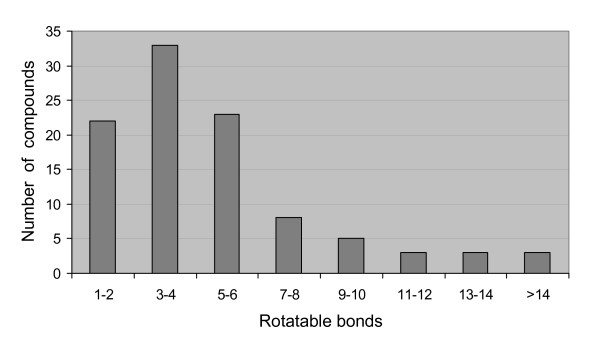
Rotatable bond distributions of 100 test chemical compounds from the NMRSHIFT collection.

**Figure 2:**
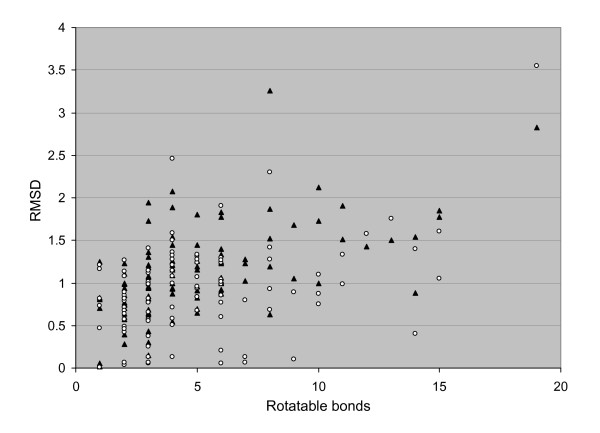
**RMSD between the best-fitted conformers (for series of up to 50) and the NMR structures versus the number of rotatable bonds for 100 chemical compounds**. ▲ for conformers generated by Multiconf-DOCK;  for conformers generated by OMEGA.

In order to evaluate our conformer generator Multiconf-DOCK, we used two validation sets. The first tests with Multiconf-DOCK and OMEGA were performed on 100 chemical compounds obtained from the NMRshift database. The distribution of rotatable bonds of these molecules is shown in Figure 1 (i.e. these compounds contain several rotatable bonds). The calculated RMSD between the best conformation generated by Multiconf-DOCK and OMEGA for series of up to 50 conformers and the predicted NMR structure versus the number of rotatable bonds for these 100 molecules are shown in Figure 2. The average RMSD between the best fitting conformer generated by Multiconf-DOCK and OMEGA and the test set was calculated to be 1.1 Å and 0.9 Å, respectively. The results obtained with both programs are satisfactory since the RMSD between all saved generated conformers was chosen to be 1.0 Å. As can be expected the ability to generate a conformation close to structures present in the NMRshift database is depending on the number of rotatable bonds (Figure 2). The RMSD between the best conformations generated with Multiconf-DOCK and NMRshift structures for 89% of the molecules with 1–9 rotatable bonds ranges from 0.1 to 1.5 Å while for 58% of the molecules with 10–15 rotatable bonds the RMSD are more than 1.5Å.

We also investigated the Multiconf-DOCK performance on the bioactive conformations of our second test set (see Methods section). We assessed the impact of changing the parameters dealing with flexibility by calculating the RMSD to experimental structures and by visual inspection. The goal here was to check whether or not Multiconf-DOCK was able to reproduce correctly the experimental bound conformation for the active molecules of our target proteins. The averaged RMSD between the ligand crystal structures and the best conformations generated by Multiconf-DOCK and OMEGA (maximum of 50 conformers per compound) for 36 PDB protein-ligand crystal structures (out of all 65 known actives) are calculated to be 1.32 Å and 1.30 Å, respectively. The best conformers generated by Multiconf-DOCK and OMEGA showed RMSD lower than 1.5 Å for 77% and 75% of the 36 X-ray structures, correspondingly. The worst RMSD of 2.45 Å was obtained for two FX inhibitors (PDB codes 1lpk for Multiconf-DOCK and 1g2l for OMEGA). A possible reason for this large RMSD could be the accuracy of the bioactive conformations. For the ligand in the 1lpk.pdb structure (resolution 2.20 Å) two conformations A and B are suggested. We computed a best RMSD value of 2.45 Å and of 1.23 Å with the conformations A and B, respectively. As recently discussed by Hawkins et al. [36], inaccuracies of some ligand structures in X-ray protein-ligand complexes have been noticed and can be due to numerous reasons, ultimately leading to high RMSD when compared to modeled ligands conformations. In the case of 1g2l.pdb with a resolution of 1.9 Å the reason may be elsewhere. The 1g2l ligand is extremely flexible with 12 rotatable bonds, and it is known that structural prediction accuracy drops as the number of rotatable bond increases. Also, generating more conformers could be valuable for such compound, additional tests performed with OMEGA and up to 100 conformers improved the result with a best RMSD value of 1.7 Å. Finally, both programs were able to retrieve the 36 bioactive conformations with an averaged RMSD value below 1.3 Å (with a maximum of 50 conformers per ligand). This RMSD value can be considered as acceptable for the generation of a "good" docked pose via rigid body docking. According to the computed RMSD values and visual analysis (see Additional file 1) both Multiconf-DOCK and OMEGA explore quite well the conformational space and are able to generate conformations that are close to the ligand co-crystal structure.

Further we applied Multiconf-DOCK to generate multi-conformer structures for the rigid docking stage 2 of our MS-DOCK VLS protocol. Our previous analysis [16] showed that an optimal balance between speed, accuracy and structural diversity of the final multi-conformer databank implies to generate about 50 conformers per compound with a RMSD value of 1.0 Å. Thus, we saved up to 50 conformers per compound for the 37970 small molecules present in our decoy library. This yielded a total of 709233 conformers (in average 19 conformers per compound) generated by Multiconf-DOCK (90 h in total) while when computations were carried out with similar parameters with OMEGA, 825250 conformers (in average 22 conformers per compound) were generated (12 h in total).

### Shape filtering with rigid-body docking by MS-DOCK

We then investigated whether MS-DOCK was able to retrieve the active compounds (see Table 1) out of a decoy drug-like library of 37970 molecules against seven protein targets: ribonuclease A (RNAse), coagulation factor X (FX), estrogen receptor (ER), CDK2 (CDK), thymidine kinase (TK), carboxypeptidase A (CBXpe) and neuraminidase (NA). Since it is well known that the performance of SBVLS methods depends on the nature of the receptor binding sites [37-39], we selected proteins with various physico-chemical properties of the binding site areas. Our validation targets can be divided into three groups depending on the pocket shape and accessibility: 1) with extremely open and flat binding site: NA with 30% degree of burial (computed as explained in [16]) and volume of about 504 Å^3 ^(computed using Q-SiteFinder [40]); 2) solvent-exposed grooves with some deep subpockets: with degree of burial <47% and volumes as follows: RNAse, CDK2 (511 Å^3^) and FX (546 Å^3^); 3) with a rather closed and deep binding pockets and high degree of burial (75%-90%): ER (572 Å^3^), CBXpe (342 Å^3^) and TK (456 Å^3^). Similarly to the binding pockets, the actives of the seven targets also display different physico-chemical properties (with two being of major importance in the present study: molecular weight and volume, see Table 1).

**Table 1:** Physico-chemical properties of the actives for the seven targets

*Protein*	*No. of heavy atoms*	*Molecular weight*	*No. of rotatable bonds*	*Volume (Å^3^)*
RNAse (8 actives)	21 – 50	322 – 791	4 – 14	253 – 613
FX (9 actives)	29 – 41	427 – 548	5 – 12	411 – 616
ER (10 actives)	29 – 45	390 – 458	7 – 15	450 – 483
CDK (10 actives)	18 – 31	241 – 449	1 – 7	250 – 447
TK (10 actives)	13 – 21	186 – 369	4 – 7	181 – 243
NA (10 actives)	17 – 25	237 – 350	4 – 10	236 – 376
CBXpe (8 actives)	12 – 16	121 – 290	2 – 5	108 – 177

**Figure 3:**
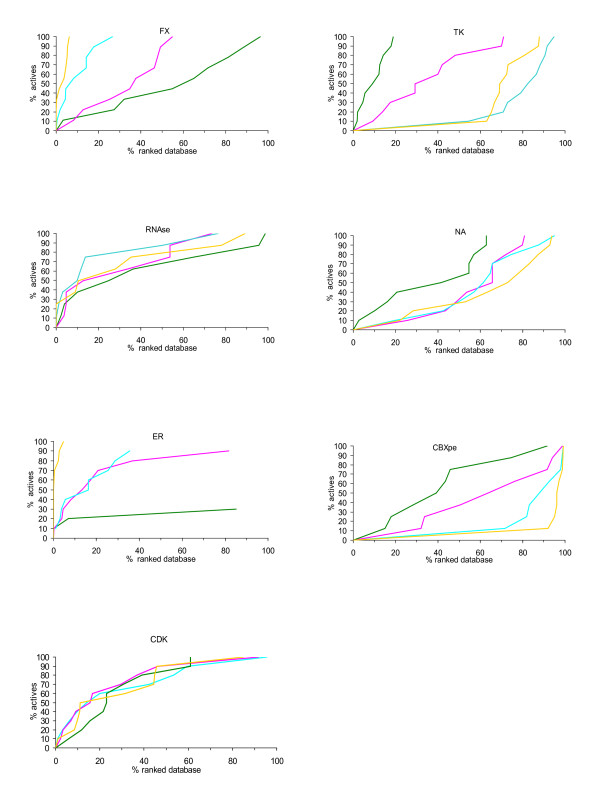
**Enrichment graphs for 7 protein targets and the 37970-compound collection**. The percentage of the ranked chemical library after multi-conformers rigid docking is plotted against the percentage of the retrieved known actives. Results are shown for the seven proteins after MS-DOCK with allowed bump overlaps of: 0.5 (in cyan), 0.6 (in magenta), 0.75 (in green). The yellow lines represent the results with FRED.

We rigidly docked the multi-conformer database generated by Multiconf-DOCK on each target and investigated how much we could reduce the input collection while retaining the active molecules. The enrichment graphs for the seven protein targets obtained by screening with MS-DOCK the library of 37970 drug-like chemical compounds with in multi-conformer states generated by Multiconf-DOCK, are presented in Figure 3. Following the MS-DOCK computations, the rigid body docking experiments with DOCK6 were performed with our optimized parameters using the contact scoring function (see Methods). Two simulations were carried out: one with a bump filter (the number of maximum allowed bumps sets to 8 which can be considered as quite permissive, allowing for some atomic clashes, as sometimes required for rigid body docking) and one without bump filter. Since no important differences were noticed, here we show only the enrichment graphs with the applied bump filter. Our preliminary tests with three different values (50 by default, 30 and 0) for penalty of each contact clash (see Methods for details) showed best performance with a contact clash penalty of 30. Thus, all present results with MS-DOCK are obtained with a clash penalty of 30 per bump.

In an attempt to better take into account the important structural/chemical differences seen in the ligands and the binding pockets, we tested 3 possible allowed bump overlaps from 0.5 to 0.75. A lower clash bump overlap parameter tolerates relatively close contacts between the ligand and the receptor atoms. One can expect that larger ligands will tend to generate more clashes, keeping in mind that the procedure involves rigid-body docking. Clearly, the tuning of the scoring/docking methods according to the nature of the binding sites/ligands can improve the performance of VLS experiments [16,41]. The results with MS-DOCK shown in Figure 3 are in agreement with this line of reasoning. Satisfactory enrichment was achieved in the case of CDK with the three values of the clash bump overlap. For the proteins RNAse, ER, and FX, displaying binging sites with a large volume and binding relatively large inhibitors (see Table 1), the best results are observed with the more permissive clash bump overlap of 0.5 (Figure 3, in cyan). In contrast, when much smaller inhibitors are active, for instance for TK, CBXpe and NA, the preferred clash bump overlaps is 0.75 (Figure 3, in green). For example we evaluated the ratio Volume of the ligand/Volume of the pocket using Q-SiteFinder [40] to be 0.95 for FX 1f0r.pdb and 0.45 for TK 1e2k.pdb. Thus, applying a clash overlap of 0.5 for large inhibitors (FX, ER, RNAse) and 0.75 for small inhibitors (TK), at least 70% of the real actives are retrieved with the MS-DOCK method in the top 30% of the ranked database after the multi-conformer rigid docking/contact scoring step. For NA and CBXpe (with ratio Volume of the ligand/Volume of the pocket of 0.44 and 0.42, respectively) we still note acceptable results with MS-DOCK. In the top 40% of the shape-scored database, 50% of the actives are retrieved.

One obvious question arises at this stage of our analysis: if no information is available about active ligands for a given target, which parameters should be used for efficient reduction of the database via rigid-body docking with MS-DOCK? In such a case we suggest a bump clash overlap of 0.6 since the results (see Figure 3, in magenta) for five targets FX, RNAse, ER, CDK and TK show an overall good enrichment with 80% of the actives retrieved in the top ~50% (enrichment factor of 1.6). Forty per cent are retrieved in the top 50% for NA and CBXpe (enrichment factor of 0.8). As final results, after applying MS-DOCK with parameters adapted to the binding pocket and the size of the ligands, the chemical library can be successfully reduced by 2- to 3-fold, thus computational time can be significantly decreased for a subsequent flexible docking step.

### Shape filtering with rigid-body docking by FRED

In order to further evaluate MS-DOCK, similar shape filtering computations were performed with FRED while the multi-conformer library was generated with OMEGA. The results for the seven proteins are shown in Figure 3 (in yellow). For the proteins FX, RNAse, ER, CDK, and CBXpe similar results were obtained with different box sizes defining the search zone. The enrichments achieved with a box extension of 4 Å around a reference ligand are shown in Figure 3. In the cases of TK and NA, the best results were obtained with a box of 2 Å around the reference ligand (Figure 3). While FRED is extremely efficient for FX and ER (retrieving the actives in the top 10% of the bank), it fails for NA, TK, and CBXpe. The shapegauss scoring function with applied "Optimization" is not able to rank properly the relatively small active compounds of TK, NA, and CBXpe.

## Discussion

Recent successful applications of SBVLS for hit discovery, lead optimization and target-based library design have been reported [15] but most of the different steps can be optimized. Here we suggest that shape-matching tools able to rapidly reduce the size of the input library are of importance not only to save CPU time but also to enhance the performance of the overall docking-scoring methods [16,20]. Along this line of reasoning, recent studies underline the need of using target specific libraries since active compounds can be obscured by the vast noise of a large database in which they are contained (see for instance Orry et al. [14]). To overcome the exponential problem inherent to flexible ligand docking methods, one can perform rigid-body docking with multiple conformer libraries as input and shape complementarity search as a first filtering step of a hierarchical procedure. Such protocol should be able to rapidly dock a large number of molecules, filtering out in a relatively "crude" way irrelevant compounds while the molecules fitting well in the binding pocket can be passed to a next, more precise, flexible docking-scoring stage. Although some commercial or proprietary packages able to perform rigid body docking are available, it is important to offer free-of-charge programs (like DOCK, Multiconf-DOCK and MS-DOCK) to academic scientists working in the field of drug discovery since funding can be rather limited in many institutions.

On the other hand, neglecting receptor flexibility in VLS protocols [42] can be problematic when dealing with some targets (i.e., induced fit [43] or enhanced flexibility at the protein-protein interfaces [44]). Recently, several studies have been published suggesting various strategies to treat receptor flexibility in VLS studies [43,45,46]. However, this is very challenging because the number of possible conformations rises exponentially with the number of rotatable bonds and the full sampling of all possible conformations is not achievable at present at the initial phase of a project when dealing with thousands of compounds. As such, many approaches implement pseudo-flexibility from the receptor side, these ones may or may not lead to better enrichment [47]. However, the purpose of MS-DOCK is to allow for a fast shape based pre-filtering step and it is not intended to directly treat the flexibility of the binding pocket. Yet, MS-DOCK can be easily applied on multiple receptor conformations generated either through simulations (for instance by normal mode analysis or molecular dynamics/Monte Carlo simulations [48]) or experimentally (several NMR or X-ray structures [21]).

Lorber and Shoichet have also [27] proposed to dock rigidly hierarchical databases containing ligands in a multiple-conformer state. One problem of using their approach is the absence of treatment of the ligand's internal energy. Therefore, this protocol can easily lead to unrealistic ligand conformations (for instance important internal ligand clashes may be present) fitting apparently well into the binding pocket while being indeed a computational artifact. This in turn increases the noise of the ranked database because some compounds that can not fit into the binding pocket are still selected and may indeed have a very favorable binding score. On the contrary, generating multiple ligand conformations within a reasonable energy window (not necessarily only the lowest energy conformations) like with Multiconf-DOCK or OMEGA prior to rigid-body docking allows for the selection of relevant ligand conformations. In [23] the authors proposed to dock multiple ligand conformers generated by random increment of 120° to the dihedral angles on a rigid receptor. In a different way, our treatment of rotatable bonds by a systematic search with increased flexibility in Multiconf-DOCK (see details in the Results section: *Multiconf-DOCK: method and implementation*) permits better exploration of the ligand conformational space but still within an allowed energy window. Our proposed Multiconf-DOCK tool was capable to generate conformations close to predicted NMR structures of small molecules (see Figure 2). For 80% of the test compounds with up to 15 rotatable bonds, the best fit to the experimental structure showed RMSD values lower than 1.5 Å. In addition, it was also of major importance to test Multiconf-DOCK for its ability to generate conformations similar with bioactive conformations (see Additional file 1) because one goal of the present study is to propose a geometric filter able to reduce the size of the input chemical library. The averaged RMSD of 1.3 Å between the best fitting predicted structures and the experimental crystal structures for the 36 actives proves that the changes that we have introduced in the parameters increasing ligand flexibility whenever appropriate. As mentioned above, we stress the attention of the readers to the fact that with the default low-energy dihedral angles implemented in DOCK5, a number of ligand conformations bound to a protein could not be reproduced [16,32]. Indeed, it is known that when a ligand binds to a protein, it is typically not in the lowest-energy conformation [49,50]. One of the factors determining high strain energies in bound ligands may be the process of unfolding, in which the intramolecular interactions between hydrophobic groups in ligands are released to increase the interactions with the protein binding site as suggested in [49].

After analysis of the conformer generation step, we evaluated the performance of MS-DOCK and FRED (ran in its shape-complementarity mode only to make the present comparison meaningful) to retrieve known actives seeded in a drug-like library of 37970 molecules for seven target proteins with various binding site geometries and properties. FRED is very efficient when large ligands are bound in a relatively large binding site as in the cases of ER, RNAse, CDK and FX. On the contrary, the FRED shape-filtering search failed when relatively small ligands are bound in the pocket (the cases of TK, CBXpe and NA). MS-DOCK showed good results for ER, RNAse, FX, CDK and TK, allowing to keep only the top 30% of the initial library. For NA and CBXpe, because the inhibitors are small and the active sites are relatively large, MS-DOCK does not perform very well, yet the results are acceptable. In such cases 50% of the ranked database should be considered for subsequent more precise flexible docking experiments. In terms of speed, MS-DOCK screened our collection containing 709233 conformations in 70 h with on average for the seven targets (2.8 conformers per sec; ~10 ligands per min), on one CPU compared to 1 ligand per min for flexible ligand docking with DOCK. Thus the MS-DOCK method is 10-fold faster than the standard flexible ligand docking with DOCK and it can speed up considerably the VLS process, allowing a reduction of at least 2-fold the subsequent flexible docking step. All computations in this study were carried out on two Linux workstations (Xeon 3.0 GHz and 1.5 Gb RAM). Averaged on the seven targets, FRED screened the database of 825250 conformations in 30 h. On average, FRED docked 8 conformers per second on the same computer and is thus 3 times faster than MS-DOCK. Yet, while FRED achieves faster rigid-body docking, MS-DOCK shows a better overall performance for the purpose of creating target specific libraries based on shape complementarity only.

## Conclusion

We have developed MS-DOCK to rapidly screen a large compound collection for the generation of "focused" libraries of reduced size containing molecules with satisfactory shape complementarity with the receptor binding pocket. MS-DOCK employs the in-house developed tool Multiconf-DOCK for multiconformer library generation. In addition, better handling of ligand flexibility as implemented in Multiconf-DOCK can easily be employed to improve the docking accuracy of flexible docking with DOCK6.

The MS-DOCK method, which is based on the widely used program DOCK (both free-of-charge for academic institutions) tends to perform better than FRED when this one is applied as geometric filters at least on our validation set. Depending on the target-binding site, MS-DOCK allows the use of a fraction of the initial databank (typically 30–50%) without compromising the performance of the protocol in retrieving actives.

We argue that multi-stage SBVLS can help to improve the speed and rate in the search of hit compounds with new scaffolds. Applying shape filtering as a first step of a structure-based screening protocol can result in the creation of target-specific libraries without decreasing the chemical diversity of the selected compounds. In this line of reasoning the MS-DOCK method can successfully be applied as a part of a hierarchical VLS procedure in order to lower the length of the computations while improving the performance of the overall procedure.

## Methods

### Preparation of the validation sets

#### Target structures

Seven protein targets were chosen with diverse binding site properties. Crystal structures of estrogen receptor (ER, PDB code 3ert, resolution 1.90 Å), thymidine kinase (TK, PDB code 1kim, resolution 2.14 Å), coagulation factor X (FX, PDB code 1f0r, resolution 2.10 Å), ribonuclease A (RNAse, PDB code 1afk, resolution 1.70 Å), neuraminidase (NA, PDB code 1b9s, resolution 2.50 Å), CDK2 (CDK, PDB code 1fvv, resolution 2.80 Å), and carboxypeptidase A (CBXpe, PDB code 1hdq, resolution 2.30 Å) were selected for the VLS experiments. For each target we retrieved from the PDB [51] up to ten X-ray holo structures with resolution from 1.20 Å to 2.80 Å. The final selection of the receptor structures for the VLS experiments was done by analysis of the binding sites in the retrieved protein-ligand complexes since upon ligand binding conformational changes of receptors can occur, from 'large-scale' loops to single side-chain movements [48]. The superimposition of the structures showed practically identical 3D structures for FX and NA. In the cases of TK and ER, for some of the X-ray structures, several residues next to the binding pockets were missing, thus we selected the best and more complete X-ray structures. Interestingly, for three of the targets, conformational differences in/close to the binding sites were present, namely for RNAse (side-chains movements), CDK (a loop movement) and CBXpe (with pocket volume depending on the bound ligands). For these three proteins we chose the most open binding pockets among the several crystal complexes available at the PDB. For all proteins the water molecules and ligands were removed from the binding sites. Hydrogen atoms were added to protein structures using the program InsightII [52]. The seven proteins and corresponding ligands structures used in this study can be downloaded from our website [53].

#### Compound library

The chemical library for our VLS experiments can be found at the RPBS web server [54] in the section FAF-Drugs [55,56]. Our testing set is based on the 2004 release of the ChemBridge Diversity set database. The 50080 molecules were first filtered in order to remove non-drug like compounds using the program FILTER version 1.0.2 [57] with a slightly modified filtering parameter file. The main parameters that were modified are: molecular weight (min/max) 100 Da/900 Da; number of carbons (min/max) 5/35; rotatable bonds (min/max) 0/20; hydrogen bond donors/acceptors (max) 8/12, sum formal charges (min/max) -2/2; XlogP (min/max) -5/6; 2D polar surface area (min/(max) 0/160 Å ^2^; and rejection of about 100 toxic/reactive functional groups. The resulting ADME/Tox filtered library contained 37970 compounds. To this collection we added the 65 active inhibitors of the seven target proteins, all in SMILES format. These molecules were transformed in 3D (single conformer) using the program OMEGA v.2.

#### Validation compounds sets for multiple conformation generation

One testing set for the validation of our Multiconf-DOCK tool (multiple conformer generation) was downloaded from the NMRSHIFT website [58,59]. This collection contains predicted 3D structure of small molecules carried out with a structure-coding scheme correlating structural features with chemical shift values. We chose randomly 130 small molecules to investigate Multiconf-DOCK and removed from this list molecules that did not contain any rotatable bonds. One hundred small chemical compounds with various numbers of rotatable bonds (see Figure 1) were finally used to investigate our multiple 3D conformers generator. The same 100 compounds were subjected to multiple conformation generation with OMEGA. We also generated a second validation set by extracting 36 ligands (out of the 65 actives of our targets) from X-ray protein-ligand structures with resolution from 1.20 Å to 2.60 Å. These two sets of 100 and 36 chemically diverse compounds were used to assess OMEGA and Multiconf-DOCK. The physico-chemical properties of both validation compounds sets were assessed via the ADME/tox filtering tool FAF-Drugs [55,56] available on the RPBS web server [54]. The ligand volumes were computed with InsightII [52].

### Shape filtering by rigid-body docking with MS-DOCK

MS-DOCK employs a rigid-body docking method as implemented in DOCK6. It aims at performing a fast "geometric" filtering selection of ligands that fit well in the binding pocket using only a surface complementarity criterion. DOCK fits the multiple rigid conformers into the rigid receptor by calculating an orientation for each conformer in the binding site, and then by evaluating the fit. We applied the sphere method of Kuntz [22] to identify pseudo-atom positions of a ligand in the receptor. DOCK uses spheres representing a negative image of the receptor-binding site and ligand heavy atom centers to rigidly orient ligands in the receptor. We used the program DMS [60] to compute the molecular surface of the receptor. The overlapping spheres within a radius of 4 Å were generated on the protein binding site surface with the program SPHGEN [61]. Sphere clusters within 6 Å to a ligand reference were retained for all our protein targets but for NA that possesses an open and flat binding site. For this target, the distance was limited to 4 Å in order to diminish possible noise most created by very large compounds artificially fitting to the very flat binding site. For orientation of the ligands in the binding sites both automated and manual match algorithms are available in DOCK6, they proceed through matching of all receptor sphere pairs to ligand's atom pairs. While the automated matching generates matches until the chosen maximum number of orientations is reached (thus equal number of orientations for all molecules), the manual matching generates only the matches which satisfy user defined distance (intra-ligand or intra-receptor distances allowed in a match) and node (numbers of atom-site point interactions needed to construct an orientation) parameters. In this way, when the manual matching is applied, ligands with more similar internal distances with the receptor site points will be prioritized. Finally a smaller number of orientations compared to the automated matching will be scored reducing computational time. In order to speed up the calculations, we applied manual match (the parameters are given in the Additional file 2) with a limit of a maximum 500 orientations.

The generated orientations of the different conformers are scored in order to evaluate the degree of fit with the receptor. In our calculations, the score measures only the steric complementarity by use of the simplest and fastest contact scoring function. The contact score procedure available in DOCK adds up the number of receptor atoms within a prescribed distance range (4.5 Å by default) defining potentially attractive interactions. If the two atoms approach close enough to clash as identified with the bump grid, then the interaction can be penalized. We tested three different allowed bump overlaps (amount of van der Waals overlap; for a value of 1 no overlap is allowed): 0.75 (default), 0.60 and 0.50. In addition three different values of contact clash penalty per atom bump were tested: 50 (default), 30 and 0. We carried out rigid-body docking on seven protein targets with multiple conformations pre-generated with Multiconf-DOCK for all molecules of the database. The key MS-DOCK input parameters can be seen in the Additional file 2.

### Generation of multiple conformations by OMEGA

The algorithm implemented in OMEGA v.2 (Openeye Scientific Software) dissects the molecules into fragments and uses fragment templates to build a seed conformation (see [57]). Next OMEGA begins torsion search with an assessment of freely rotatable bonds. Conformers are generated and are associated with a strain energy evaluated using the Merck molecular force field. The termination criteria to generate conformers can be the total number of generated conformers decided by the user. One key parameter is the ewindow value which defines the strain energy range within which conformers are considered as acceptable (the default ewindow was set to 25.0 kcal/mol). Next to ewindow, the parameter RMSD plays also a major role as it sets the minimum root mean square deviation of coordinates below which two conformers are considered to be identical. In our computations and following results from the literature, we generated a maximum of 50 conformations per molecule and used an ewindow of 25.0 kcal/mol and an RMSD cutoff of 1.0 Å [28]. We used this large RMSD value to ensure adequate conformational sampling and important structural diversities, while the large ewindow value was chosen because in many cases the conformation adopted by small compounds co-crystallized into a protein-binding site can be relatively far from global minimum energy conformations [49,50].

### Shape filtering by rigid-body docking with FRED

FRED 2.1 [26] (Openeye Scientific Software) was used in this study to dock the OMEGA pre-generated multi-conformer library mentioned above. FRED 2.1 strategy is to exhaustively dock/score all possible positions of each ligand in the binding site. The exhaustive search is based on rigid rotations and translations of each conformer within the binding site defined by a box created by the users. FRED filters the poses ensemble by rejecting the ones that clash with the protein or that do not have enough contacts with the receptor. The final poses can then be scored or re-scored using one or more scoring functions. In this study we decided to select the smooth shape-based Gaussian scoring function (shapegauss) to evaluate the shape complementarity between each ligand and the binding pocket. The reason for this choice is that this protocol is then comparable to the MS-DOCK rigid-body docking protocol described above while the search approach and the scoring differ. We used the default FRED protocol except for the size of the box defining the binding sites. In an attempt to optimize the docking-scoring performance we performed exhaustive docking with shapegauss applying the "Optimization" mode. The "Optimization" mode involves a systematic solid body optimization of the top ranked poses from the exhaustive docking. We explored 3 different boxes: for each protein three different simulations were carried out with an added value of 6, 4 (by default) or 2 Å around the reference ligand.

## Authors' contributions

NS contributed to development and validation of the method. DL contributed to the analysis and data interpretation. BOV and MAM designed MS-DOCK. MAM developed Multiconf-DOCK and coordinated the study. All authors contributed to the writing, read and approved the final manuscript.

## Supplementary Material

Additional file 1 Predicted conformations of five small molecules generated by Multiconf-DOCK and OMEGA. Predicted conformations generated by Multiconf-DOCK and OMEGA superimposed onto the experimental structure. Click here for file

Additional file 2 Input parameters for MS-DOCK. Input parameters for Multiconf-DOCK, rigid docking with DOCK6 and GRID. Click here for file
