# Supplementary material for: MS-DOCK: Accurate multiple conformation generator and rigid docking protocol for multi-step virtual ligand screening
Source: BMC Bioinformatics. 2008 Apr 10;9:184. doi: 10.1186/1471-2105-9-184 (PMC2373571; doi:10.1186/1471-2105-9-184)
Supplement: Additional file 1 — Predicted conformations of five small molecules generated by Multiconf-DOCK and OMEGA. Predicted conformations generated by Multiconf-DOCK and OMEGA superimposed onto the experimental structure. [file 1471-2105-9-184-S1.pdf]

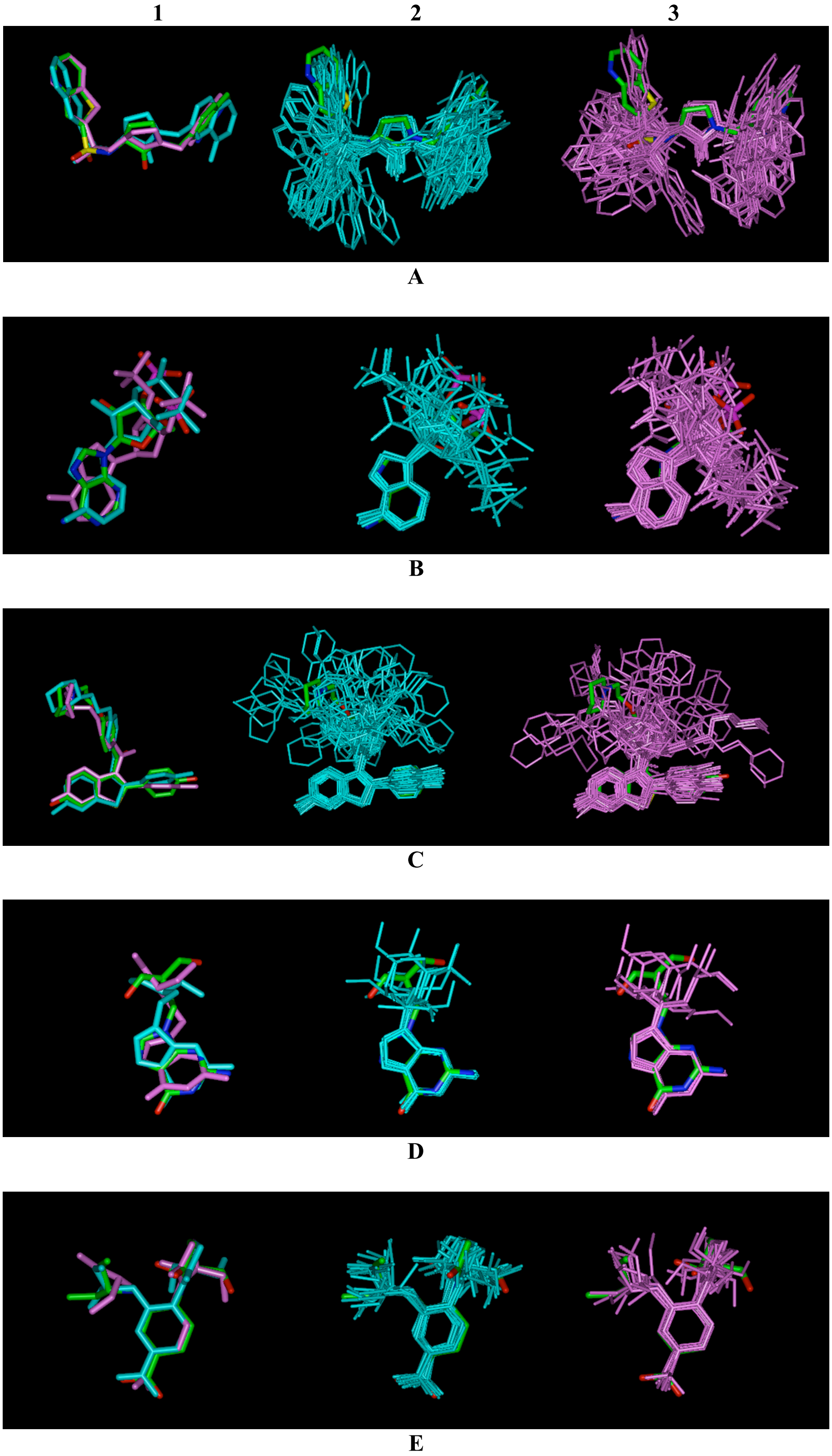

Figure 1

Predicted versus experimental structures. Panels A, B, C, D and E refer to the five proteins of the validation set. Within each panel, 1 shows the superimposition of the experimental ligand structure (all atom colors) and the best conformers generated by OMEGA (cyan) and Multiconf-DOCK (magenta), 2 shows up to 50 predicted conformations generated by OMEGA (cyan) superimposed onto the experimental structure , and 3 shows up to 50 predicted conformations generated by Multiconf-DOCK (magenta) superimposed onto the experimental structure. Panel A: FX, 1f0r.pdb; Panel B: RNAse 1o0f.pdb; Panel C: ER 1err.pdb; Panel D: TK 1ki3.pdb; Panel E: NA 1b9v.pdb.
