# Supplementary material for: MS-DOCK: Accurate multiple conformation generator and rigid docking protocol for multi-step virtual ligand screening
Source: BMC Bioinformatics. 2008 Apr 10;9:184. doi: 10.1186/1471-2105-9-184 (PMC2373571; doi:10.1186/1471-2105-9-184)
Supplement: Additional file 2 — Input parameters for MS-DOCK. Input parameters for Multiconf-DOCK, rigid docking with DOCK6 and GRID. [file 1471-2105-9-184-S2.pdf]

INPUT PARAMETER FILE   for Multiconf-DOCK

```
ligand_atom_file                                              input.mol2
ligand_outfile_prefix                                         output_50conf
limit_max_ligands                                             no/yes
max_ligands                                                   10000
write_conformations                                           yes
initial_skip                                                  0
max_conformations                                             50
calculate_rmsd                                                yes/no
use_rmsd_reference_mol                                        yes/no
rmsd_reference_filename                                       Xray_tocompare.mol2
rmsd_window                                                   1.0
energy_window                                                 25.0
min_anchor_size                                               5
number_confs_for_next_growth                                  50
use_internal_energy                                           yes
internal_energy_att_exp                                       6
internal_energy_rep_exp                                       12
internal_energy_dielectric                                    4
atom_model                                                    all
vdw_defn_file                                        vdw_AMBER_parm99.defn
flex_defn_file                                       flex_Multiconf-DOCK.defn
flex_drive_file                                      flex_drive_Multiconf-DOCK.tbl
```

INPUT PARAMETER FILE for Rigid Docking with DOCK6

```
ligand_atom_file                                                   input.mol2
ligand_outfile_prefix                                         output_RigidDocking_DOCK6
limit_max_ligands                                                  no
read_mol_solvation                                                 no
write_orientations                                                 no
write_conformations                                                no
skip_molecule                                                      no
calculate_rmsd                                                     no
rank_ligands                                                       no
num_scored_conformers_written                                      1
orient_ligand                                                      yes
automated_matching                                                 no
distance_tolerence                                                 0.25
distance_minimum                                                   2.0
nodes_minimum                                                      3
nodes_maximum                                                      10
receptor_site_file                                                 ../2_site/site.sph
max_orientations                                                   500
critical_points                                                    no
chemical_matching                                                  no
use_ligand_spheres                                                 no
flexible_ligand                                                    no
bump_filter                                                        yes
bump_grid_prefix                                                   ../3_grid/grid_06
max_bumps                                                          8
score_molecules                                                    yes
contact_score_primary                                              yes
contact_score_secondary                                            yes
contact_score_cutoff_distance                                      4.5
contact_score_clash_overlap                                        0.6
contact_score_clash_penalty                                        30.0
contact_score_grid_prefix                                          ../3_grid/grid_06
minimize_ligand                                                    no
atom_model                                                         all
vdw_defn_file    /usr/local/programs/DOCK/dock6/parameters/vdw_AMBER_parm99.defn
flex_defn_file   /usr/local/programs/DOCK/dock6/parameters/flex.defn
flex_drive_file  /usr/local/programs/DOCK/dock6/parameters/flex_drive.tbl
```

INPUT PARAMETER FILE for GRID

```
compute_grids                  yes
grid_spacing                   .3
output_molecule                no
contact_score                  yes
contact_cutoff_distance        4.5
chemical_score                 no
energy_score                   no
energy_cutoff_distance         10
atom_model                     a
attractive_exponent            6
repulsive_exponent             9
distance_dielectric            yes
dielectric_factor              4
bump_filter                    yes
bump_overlap                   0.6
receptor_file                  ../1_struct/receptor.mol2
box_file                       box.pdb
vdw_definition_file  /usr/local/programs/DOCK/dock6/parameters/vdw_AMBER_parm99.defn
chemical_definition_file       /usr/local/programs/DOCK/dock6/parameters/chem.defn
score_grid_prefix              grid_06
```
